# Differentiation of glaucomatous optic discs with different appearances using optic disc topography parameters: The Glaucoma Stereo Analysis Study

**DOI:** 10.1371/journal.pone.0169858

**Published:** 2017-02-08

**Authors:** Masaki Tanito, Koji Nitta, Maki Katai, Yasushi Kitaoka, Yu Yokoyama, Kazuko Omodaka, Toru Nakazawa

**Affiliations:** 1 Division of Ophthalmology, Matsue Red Cross Hospital, 200 Horo-machi, Matsue, Shimane, Japan; 2 Department of Ophthalmology, Shimane University Faculty of Medicine, 89–1 Enya, Izumo, Shimane, Japan; 3 Department of Ophthalmology, Fukui-ken Saiseikai Hospital, Fukui, Japan; 4 Department of Ophthalmology, Sapporo Medical Center Nippon Telegraph and Telephone East Corporation, Sapporo, Japan; 5 Department of Ophthalmology, St. Marianna University School of Medicine, Kanagawa, Japan; 6 Department of Ophthalmology, Tohoku University Graduate School of Medicine, Sendai, Japan; Bascom Palmer Eye Institute, UNITED STATES

## Abstract

The Glaucoma Stereo Analysis Study (GSAS) is a multicenter collaborative study of the characteristics of glaucomatous optic disc morphology using a stereo fundus camera. Using GSAS dataset, the formulas for predicting different glaucomatous optic disc appearances were established. The GSAS dataset containing three-dimensionally-analyzed optic disc topographic parameters from 187 eyes with primary open-angle glaucoma was assessed with discrimination analyses to obtain formulas predictive of glaucomatous optic disc appearances: focal ischemic (FI); generalized enlargement (GE), myopic glaucomatous (MY), and senile sclerotic (SS). Using 38 optic disc parameters-substituted discrimination analyses with a stepwise forward-selection method, six parameters (temporal and nasal rim-disc ratios, mean cup depth, height variation contour, disc tilt angle, and rim decentering absolute) were selected into the formulas. The area under the receiver operating characteristic curves for predicting the four disc types with established formulas were 0.88, 0.91, 0.93, and 0.86 for FI, MY, SS, and GE, respectively. Age, visual acuity, refractive error, glaucoma (normal or high-tension glaucoma), and baseline intraocular pressure differed significantly among the four optic disc types, suggesting the appearances represent different clinical glaucoma phenotypes. Using six optic disc topographic parameters obtained by stereo fundus camera, the GSAS classification formulas predicted and quantified each component of different optic disc appearances in each eye and provided a novel parameter to describe glaucomatous optic disc characteristics.

## Introduction

Glaucoma is a leading cause of irreversible blindness worldwide[1] including Japan[2]. The presence of optic disc damage resulting from loss of retinal ganglion cells (RGCs) and RGC axons characterize glaucoma[3]; thus, morphologic detection of glaucomatous optic neuropathy (GON) by various modalities is essential for diagnosing this pathology. Since GON accompanies the changes in the “depth” parameters of optic disc morphology, i.e., excavation of optic disc cupping, ophthalmoscopy or subjective examination on a single fundus photograph monoscopically might underestimate optic disc cupping and glaucoma severity[4, 5]. Topographic analysis of optic discs using a simultaneous stereo fundus camera, a noninvasive, noncontact imaging technique that does not require pupillary dilation, has been reported to have excellent reproducibility and interexaminer consistency[6], making it a promising tool to objectively assess morphologic changes in GON. The Glaucoma Stereo Analysis Study (GSAS) is a multicenter collaborative study in which this technique is used to assess various optic disc morphologic parameters in Japanese patients with primary open-angle glaucoma (POAG) as reported in the study’s initial dataset[7].

The patterns and progression of visual field defects and the prevalence of risk factors, e.g., intraocular pressure (IOP) and refractive error, vary among patients with glaucoma[8, 9]. Nicolela and Drance proposed dividing various GONs into four subgroups: focal ischemic (FI), generalized enlargement (GE), myopic glaucomatous (MY), and senile sclerotic (SS), based on the optic disc appearance[10]. Patients with different optic disc appearances, selected only by assessment of fundus photographs, had different demographic characteristics, prevalence rates of certain systemic and ocular risk factors, IOP levels, and patterns of visual field damage[3, 10–18]. Thus, classification of GONs by optic disc appearance represents different pathologic mechanisms of glaucoma and might facilitate more accurate diagnoses and better disease management.

Glaucoma specialists subjectively classify the optic disc appearance[3, 10–18]; therefore, establishment of an objective classification is important to generalize the theory of Nicolela and Drance. In the current study, using optic disc parameters obtained from the GSAS, the formulas for predicting different optic disc appearances were established (the GSAS classification) and the correlations between grader-classified (i.e., FI, GE, MY, and SS) and formula-predicted (i.e., pFI, pGE, pMY, and pSS) optic disc types were assessed.

## Subjects and methods

### Subjects

This study adhered to the tenets of the Declaration of Helsinki. The institutional review boards of Shimane University Hospital, Fukui-ken Saiseikai Hospital, Sapporo Teishin Hospital, St. Marianna University School of Medicine, and Tohoku University Graduate School of Medicine reviewed and approved the research. One hundred and eighty-seven eyes of 187 patients with POAG were recruited from five institutions including Shimane University Hospital, Fukui-ken Saiseikai Hospital, Sapporo Teishin Hospital, Hospital of St. Marianna University School of Medicine, and Tohoku University Hospital. Written informed consent was obtained from the subjects, otherwise, based on the regulations of the Japanese Guidelines for Epidemiologic Study issued by the Japanese Government, the study protocols did not require the each patient provide written informed consent, instead the protocol was posted at the outpatient clinic to notify the study to the participants. The inclusion and exclusion criteria, methods of ophthalmic examinations, diagnosis of POAG, and rules for data collection were reported previously[7].

In the current study, the following demographic and clinical parameters were extracted from the GSAS database: age, sex, best-corrected visual acuity (BCVA) that was converted to the logarithm of the minimum angle of resolution units, refractive error, glaucoma type (normal-tension glaucoma [NTG] or high-tension glaucoma [HTG]), untreated baseline IOP, IOP and number of glaucoma medications at the time of the fundus camera examination, visual field mean deviation (MD) and pattern standard deviation (PSD), MD slope (decibels/year), and the self-reported prevalence of systemic hypertension, diabetes, and hyperlipidemia. The IOP was measured by Goldmann applanation tonometry. The MD and PSD were measured using the Humphrey Visual Field Analyzer Swedish Interactive Thresholding Algorithm central 30–2 or 24–2 program (Carl Zeiss Meditec, Dublin, CA). The MD slope was calculated from the MD values recorded at least six times during a minimum of 3 years. The eyes that never had untreated and treated IOPs of 19 mmHg were considered to have NTG and other eyes were considered to have HTG. The demographic data of the subjects are shown in Table 1.

**Table 1:** Patient demographic data (n = 187) and summary of six optic disc parameters.

	Mean ± SD or n (%)	95% CI or n (%)
Age, year	61.4 ± 9.4	60.0–62.7
Sex	male, 100 (53.5)	female, 87 (46.5)
BCVA(logMAR)	-0.07 ± 0.08	-0.06–-0.08
Spherical equivalent refractive error (D)	-3.38 ± 3.75	-2.84–-3.91
Glaucoma type	NTG, 151 (80.8)	HTG, 36 (19.3)
Baseline IOP (mmHg)	16.9 ± 4.3	16.3–17.6
IOP on the test day (mmHg)	13.6 ± 2.6	13.2–13.9
No. glaucoma medications	1.3 ± 1.0	1.2–1.5
MD, dB	-4.71 ± 3.26	-4.23–-5.18
PSD, dB	8.08 ± 4.18	7.48–8.68
MD slope, dB/year	-0.12 ± 0.38	-0.07–-0.18
Systemic hypertension	no, 139 (74.3)	yes, 48 (25.7)
Diabetes	no, 142 (75.9)	yes, 45 (24.1)
Hyperlipidemia	no, 161 (86.1)	yes, 26 (13.9)
Rim-disc ratio of section 1 (temporal 90°)	0.069 ± 0.046	0.063–0.076
Rim-disc ratio of section 4 (nasal 90°)	0.197 ± 0.076	0.186–0.208
Mean cup depth, mm	0.204 ± 0.086	0.192 ± 0.217
Height variation contour, mm	0.579 ± 0.264	0.541–0.617
Disc tilt angle, degree	10.5 ± 12.5	8.7–12.3
Rim decentering absolute value	0.445 ± 0.271	0.405–0.484

SD, standard deviation; 95% CI, 95% confidence interval; BCVA, best-corrected visual acuity; NTG, normal tension glaucoma; HTG, high tension glaucoma; IOP, intraocular pressure; MD, visual field mean deviation; PSD, visual field pattern standard deviation; logMAR, logarithm of the minimum angle of resolution; dB, decibels; D, diopters.

### Optic disc topography

Stereo fundus images of the optic nerve head (ONH) were obtained using a stereo fundus camera (nonmyd WX, Kowa Company, Ltd., Aichi, Japan) that produces nonmydriatic fundus stereographs and simultaneous right and left parallactic images using one optical system to handle light paths in two directions[6]. The built-in software (VK-2 WX, prototype version, Kowa Company, Ltd.) automatically calculates the ONH morphologic parameters based on manually set contour lines for the ONH disc and cup, which in this study were determined by one of the authors (M.T.) while viewing the images stereoscopically. According to the recommendations of the Japan Glaucoma Society Guidelines for Glaucoma[19], the disc contour was delineated by the inner margin of Elschnig’s scleral ring, and the cup contour was delineated by the outer cup margin, which was indicated by the bending of the ONH vessels at the rim. The observer determined several points on the contour (typically 8–14), and the contour line then was generated automatically by software spline interpolation. Excellent intra- and inter-observer agreements of contour delineation with software assisted optic nerve head analysis was reported previously [5]. Thirty-five parameters calculated using the commercially available VK-2 WX software included the vertical cup-to-disc (C/D) ratio, upper rim width, lower rim width, cup area, disc area, rim area, C/D area ratio, rim-to-disc (R/D) area ratio, sectional R/D ratio (section 1, temporal 90°; section 2, superior-temporal 45°; section 3, superior-nasal 45°; section 4, nasal 90°; section 5, inferior-nasal 45°; and section 6, inferior-temporal 45°), cup volume, disc volume, rim volume, mean cup depth, maximal cup depth, height variation contour, and disc damage likelihood scale stage[20, 21]. The depth and volume values were calculated based on the disparity between the right and left images of the stereo image pair with a stereo matching technique, with correction for magnification by a modified Littman’s method using the refractive error and corneal curvature of each eye. For the GSAS, we also defined three novel parameters including the disc tilt angle, rim decentering, and the absolute value of rim decentering. Rim decentering was calculated using the following formula:

rim decentering=(superotemporal rim area−inferotemporal rim area)/(superotemporal rim area+inferotemporal rim area)

This value can be between -1 (rim thinning at the superotemporal area without rim thinning at the inferotemporal area) and 1 (rim thinning at the inferotemporal area without rim thinning at the superotemporal area); 0 indicates either equally thinned rims at both the supero- and infero-temporal rims or no rim thinning at both rims. The absolute values for rim decentering also were determined. The disc tilt angle was defined as the degree of the angle between the horizontal plane and the line drawn from the temporal to the nasal disc edge, passing through the center of the ONH. The details of the 38 parameters were described previously[7].

### Classification of optic disc appearances by graders

As reported previously[22], three independent graders (T.N., K.O., and Y.Y.) classified each optic disc appearance into four different types according to the proposal of Nicolela and Drance[10]: an FI disc with localized tissue loss at the superior or inferior poles and a relatively intact neuroretinal rim elsewhere; a GE disc characterized by a diffusely enlarged round cup and lack of localized defects of the neuroretinal rim; a MY disc that had a tilted appearance and temporal crescent peripapillary atrophy (PPA), excluding discs with degenerative myopia; and an SS disc with a saucerized shallow cup and diffuse neuroretinal rim tissue loss accompanied by surrounding PPA and choroidal sclerosis. Discs with features of multiple (mixed) disc types were assigned to the most prominent type. The optic disc appearances classified were matched 125 eyes (66.8%) among 3 graders initially, and, therefore, the optic disc types in the other 62 eyes were determined ultimately by discussion among the three graders. Using Cohen’s kappa statistics, the agreement between graders was considered moderate to substantial when calculated as kappa = 0.5164 between graders A and B, kappa = 0.7112 between graders A and C, and kappa = 0.6321 between graders B and C. As a result, the 187 eyes were classified into FI (34 eyes, 18.2%), GE (38 eyes, 20.3%), MY (96 eyes, 51.3%), or SS (19 eyes, 10.2%)[22]. In each optic disc type, using Fleiss’ kappa statistics, the agreement of the initial classification among the three graders was substantial for FI (kappa = 0.6414), GE (kappa = 0.6286), and MY (kappa = 0.6736) but relatively poor for SS (kappa = 0.3296).

### Statistical analysis

The data are expressed as the means ± standard deviations and analyzed using JMP statistical software version 11.00 (SAS Institute, Inc., Cary, NC). To generate prediction formulas for each optic disc appearance, discrimination analysis was performed in which the four grader-classified optic disc appearances were substituted as categories, and all 38 optic disc parameters were substituted as covariates; a stepwise forward-selection method was used to detect the significant parameters predictive of the optic disc appearances. The coincidence between the optic disc appearances classified by graders as FI, GE, MY, or SS and predicted by the generated formulas as pFI, pGE, pMY, or pSS was assessed by the receiver operating characteristic curves (ROCs) and the areas under them (AUC). For this purpose, the optic disc type with the highest probability provided by the formulas was regarded as each eye’s optic disc appearance. Significant optic disc parameters selected by discriminant analysis and various demographic parameters were compared among the four predicted optic disc appearances by one-way analysis of variance followed by a comparison between each pair of two types of optic disc appearances using the post-hoc Student t-test for comparison of continuous variables and the chi-square test for comparison of categorical variables. Based on Bonferroni’s method for correction of multiple comparisons, P<0.0083 and P<0.0017 were considered as significance levels of 5% and 1%, respectively, in the post-hoc test. Based on the mixing rate of four optic disc appearances, the optic discs were classified further into three categories. The single type was defined as an optic disc with one of four elements (i.e., pFI, pGE, pMY, or pSS) with a probability of 60% or more; the mixed type was defined as an optic disc of which none of element with a probability of 60% or more, but either pair of the top two elements have a probability of 80% or more; and the unclassifiable type was defined as an optic disc other than the single and mixed types.

## Results

**Fig 1:**
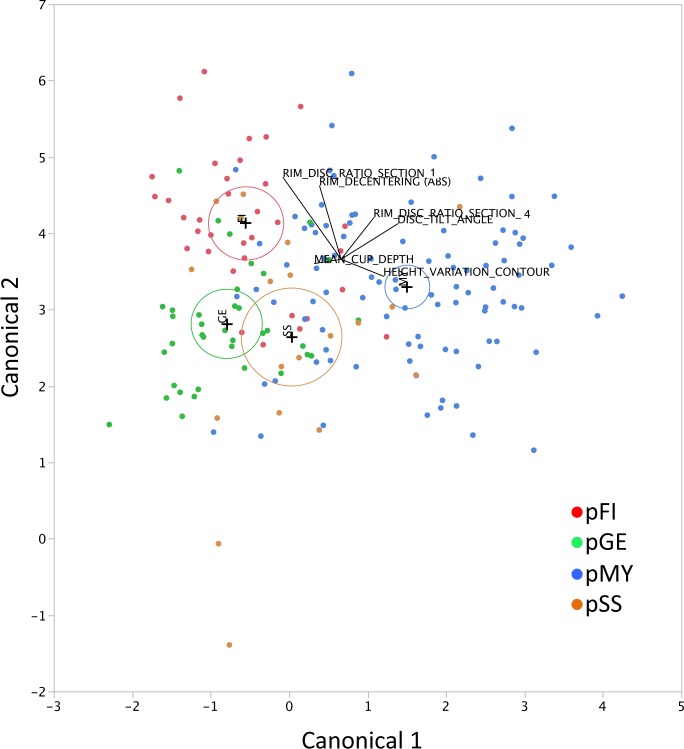
The canonical plot that are used to discriminate various optic disc appearances by the discriminant formulas. The biplot axes are the first two canonical variables that provide maximal separation among the groups. A plus (+) marker corresponds to the multivariate mean of each group. A circle around the plus marker corresponds to a 95% confidence ellipse for each mean. If two groups differ significantly, the confidence ellipses tend to not intersect. The labeled rays show the directions of the covariates in the canonical space. pFI, predicted focal ischemic; pGE, predicted generalized enlargement; pMY, predicted myopic glaucomatous; and pSS, predicted senile sclerotic.

**Fig 2:**
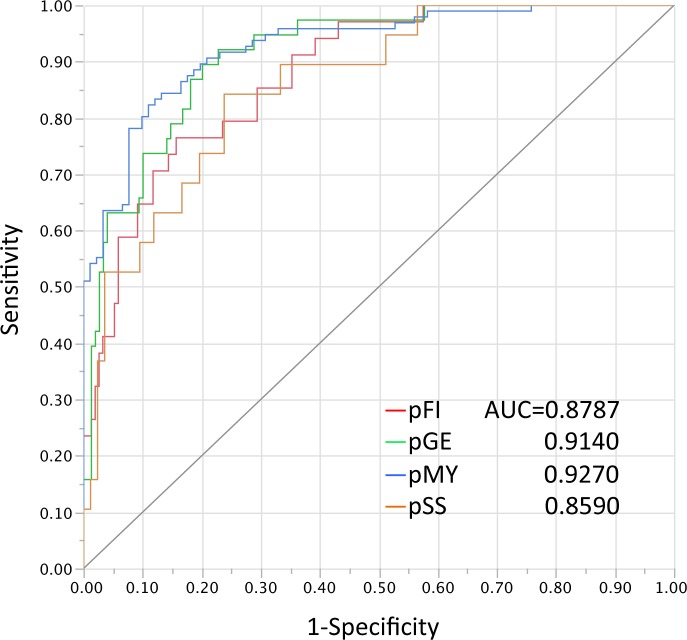
The receiver operating characteristic curves that are used to predict optic disc appearances by the discriminant formulas. pFI, predicted focal ischemic; pGE, predicted generalized enlargement; pMY, predicted myopic glaucomatous; pSS, predicted senile sclerotic; and AUC, area under the receiver operating characteristic curves.

By discrimination analyses with a stepwise variable-selection method, among 38 optic disc parameters, six parameters, i.e., the rim-disc ratio of section 1 (temporal 90°), rim-disc ratio of section 4 (nasal 90°), mean cup depth, height variation contour, disc tilt angle, and rim decentering absolute values were selected for the GSAS classification formulas (Table 1). The established discrimination formulas for predicting each optic disc appearance are shown in S1 File. When the optic disc type with the highest probability was considered in the optic disc appearance in each eye, the 187 eyes were classified as follows: 46 (24.6%) eyes with pFI, 36 (19.3%) eyes with pGE, 70 (37.4%) eyes with pMY, and 35 (18.7%) eyes with pSS. The two-dimensional canonical plot showing the distribution of each predicted optic disc appearance that provided the maximal separation among the groups is shown in Fig 1. On the canonical plot, the 95% confidence ellipses were well separated from each other, although partial overlap of the ellipses was seen between the pGE and pSS groups (Fig 1). In the ROCs, the AUC for correct classification of each optic disc type were calculated to be between 0.8590 (for pSS) and 0.9270 (for pMY) (Fig 2).

The six selected optic disc parameters differed significantly among the four predicted optic disc types (Table 2). Among the various demographic parameters, age, BCVA, refractive error, glaucoma type (NTG or HTG), and baseline IOP differed significantly among the four predicted optic disc types (Table 3).

**Table 2:** Comparisons of six optic disc parameters among four optic disc types predicted by the Glaucoma Stereo Analysis Study classification.

	pFI	pGE	pMY	pSS	P value†
N (%)					
	46 (24.6)	36 (19.3)	70 (37.4)	35 (18.7)	
Rim-disc ratio of section 1 (temporal 90°)					
Mean ± SD	0.120 ± 0.033	0.074 ± 0.031	0.042 ± 0.033	0.053 ± 0.038	<0.0001¶
95% CI	0.110–0.130	0.063–0.085	0.034–0.050	0.041–0.064	
P values‡, against pGE	<0.0001**	—	—	—	
against pMY	<0.0001**	<0.0001**	—	—	
against pSS	<0.0001**	0.0076*	0.1311	—	
Rim-disc ratio of section 4 (nasal 90°)					
Mean ± SD	0.185 ± 0.064	0.134 ± 0.047	0.246 ± 0.067	0.181 ± 0.072	<0.0001¶
95% CI	0.167–0.204	0.118–0.150	0.230–0.262	0.157–0.206	
P values‡, against pGE	0.0003**	—	—	—	
against pMY	<0.0001**	<0.0001**	—	—	
against pSS	0.7794	0.0018*	<0.0001**	—	
Mean cup depth, mm					
Mean ± SD	0.192 ± 0.045	0.300 ± 0.062	0.170 ± 0.067	0.190 ± 0.112	<0.0001¶
95% CI	0.179–0.205	0.280–0.321	0.154–0.186	0.151–0.228	
P values‡, against pGE	<0.0001**	—	—	—	
against pMY	0.1072	<0.0001**	—	—	
against pSS	0.8948	<0.0001**	0.1817	—	
Height variation contour, mm					
Mean ± SD	0.484 ± 0.155	0.413 ± 0.185	0.774 ± 0.209	0.487 ± 0.311	<0.0001¶
95% CI	0.438–0.530	0.351–0.476	0.724–0.823	0.380–0.593	
P values‡, against pGE	0.1453	—	—	—	
against pMY	<0.0001**	<0.0001**	—	—	
against pSS	0.9497	0.1542	<0.0001**	—	
Disc tilt angle, degrees					
Mean ± SD	8.04 ± 6.37	2.77 ± 7.82	22.14 ± 8.10	-1.84 ± 9.56	<0.0001¶
95% CI	6.15–9.93	0.12–5.42	20.21–24.07	-5.13–1.44	
P values‡, against pGE	0.0033*	—	—	—	
against pMY	<0.0001**	<0.0001**	—	—	
against pSS	<0.0001**	0.0157	<0.0001**	—	
Rim decentering absolute value					
Mean ± SD	0.578 ± 0.272	0.375 ± 0.250	0.413 ± 0.277	0.403 ± 0.226	0.0013¶
95% CI	0.497–0.959	0.291–0.459	0.347–0.479	0.326–0.480	
P values‡, against pGE	0.0006**	—	—	—	
against pMY	0.0011**	0.4768	—	—	
against pSS	0.0032*	0.6528	0.8499	—	

P values (†) are calculated among four types of optic disc appearances by one-way analysis of variance (ANOVA) (†) followed by comparison between each pair of two types of optic disc appearances using the post-hoc Student t-test (‡). The ¶ indicates significance levels of 5% and 1% by one-way ANOVA, respectively. In the post-hoc test, based on Bonferroni 's method to correct multiple comparisons, P<0.0083 and P<0.0017 are considered to be significance levels of 5% (*) and 1% (**), respectively.

The pFI, pGE, pMY, and pSS indicate respective types of optic disc appearances predicted by the GSAS classification formulas.

SD, standard deviation; 95% CI, 95% confidence interval; pGE, formula-predicted focal ischemia; pMY, formula-predicted myopic glaucomatous; pSS, formula-predicted senile sclerotic; pGE, formula-predicted generalized enlargement.

**Table 3:** Comparisons of various demographic parameters among four optic disc types predicted by the Glaucoma Stereo Analysis Study classification.

	pFI	pGE	pMY	pSS	P value†
N (%)					
	46 (24.6)	36 (19.3)	70 (37.4)	35 (18.7)	
Age, years					
Mean ± SD	63.3 ± 7.8	61.9 ± 10.8	58.0 ± 9.0	65.0 ± 8.5	0.0007¶
95% CI	61.0–65.6	58.2–65.6	55.9–60.2	62.1–67.9	
P values‡, against pGE	0.4756	—	—	—	
against pMY	0.0022*	0.0372	—	—	
against pSS	0.4099	0.1486	0.0002	—	
Sex					
male, n (%)	19 (41.3)	24 (66.7)	40 (57.1)	17 (48.6)	0.1100
female, n (%)	27 (58.7)	12 (33.3)	30 (42.9)	18 (51.4)	
BCVA(logMAR)					
Mean ± SD	-0.07 ± 0.07	-0.08 ± 0.09	-0.08 ± 0.08	-0.03 ± 0.09	0.0132§
95% CI	-0.09–-0.05	-0.10–-0.05	-0.10–-0.06	-0.06–+0.01	
P values‡, against pGE	0.9333	—	—	—	
against pMY	0.7805	0.8673	—	—	
against pSS	0.0091	0.0110	0.0022*	—	
Refractive error, SE (D)					
Mean ± SD	-1.54 ± 3.70	-1.74 ± 3.00	-5.14 ± 2.97	-3.97 ± 4.19	<0.0001¶
95% CI	-2.64–-0.44	-2.75–-0.72	-5.85–-4.43	-5.41–-2.53	
P values‡, against pGE	0.7948	—	—	—	
against pMY	<0.0001**	<0.0001**	—	—	
against pSS	0.0018*	0.0065*	0.1000	—	
Glaucoma type					
NTG, n (%)	42 (91.3)	24 (66.7)	60 (85.7)	25 (71.4)	0.0123§
HTG, n (%)	4 (8.7)	12 (33.3)	10 (14.3)	10 (28.6)	
Baseline IOP (mmHg)					
Mean ± SD	15.5 ± 4.1	18.0 ± 4.5	17.2 ± 3.9	17.3 ± 4.7	0.0423§
95% CI	14.2–16.7	16.5–19.5	16.3–18.1	15.7–18.9	
P values‡, against pGE	0.0078*	—	—	—	
against pMY	0.0331	0.3524	—	—	
against pSS	0.0534	0.4943	0.8901	—	
IOP on the test day (mmHg)					
Mean ± SD	13.2 ± 2.6	14.2 ± 3.0	13.5 ± 2.4	13.6 ± 2.8	0.3720
95% CI	12.4–14.0	13.2–15.2	12.9–14.0	12.7–14.6	
No. of glaucoma medication					
Mean ± SD	1.2 ± 0.8	1.3 ± 1.0	1.3 ± 1.0	1.6 ± 0.9	0.2071
95%CI	1.0–1.4	1.0–1.7	1.1–1.5	1.3–2.0	
MD, dB					
Mean ± SD	-4.01 ± 3.23	-4.34 ± 3.26	-4.88 ± 3.24	-5.66 ± 3.21	0.1268
95% CI	-4.97–-3.06	-5.44–-3.24	-5.65–-4.10	-6.76–-4.56	
PSD, dB					
Mean ± SD	7.43 ± 4.33	6.95 ± 3.65	8.59 ± 4.13	9.08 ± 4.37	0.0766
95% CI	6.14–8.71	5.71–8.18	7.61–9.58	7.58–10.58	
MD slope, dB/year					
Mean ± SD	-0.19 ± 0.42	-0.07 ± 0.40	-0.11 ± 0.34	-0.11 ± 0.41	0.5544
95% CI	-0.31–-0.06	-0.21–+0.06	-0.19–-0.03	-0.25–+0.04	
Systemic hypertension					
no, n (%)	36 (78.3)	31 (86.1)	50 (71.4)	22 (62.9)	0.1168
yes, n (%)	10 (21.7)	5 (13.9)	20 (28.6)	13 (37.1)	
Diabetes					
no, n (%)	37 (80.4)	30 (83.3)	48 (68.6)	27 (77.1)	0.2974
yes, n (%)	9 (19.6)	6 (16.7)	22 (31.4)	8 (22.9)	
Hyperlipidemia					
no, n (%)	37 (80.4)	32 (88.9)	61 (87.1)	31 (88.6)	0.6551
yes, n (%)	9 (19.6)	4 (11.1)	9 (12.9)	4 (11.4)	

P values (†) are calculated among four types of optic disc appearances by one-way analysis of variance (ANOVA) followed by comparison between each pair of two types of optic disc appearances using the post-hoc Student t-test (‡) for the continuous variables, and by the chi-square test for the categorical variables. The § and ¶ indicate significance levels of 5% and 1%, respectively, by one-way ANOVA or chi-square test. In the post-hoc test, based on Bonferroni 's method to correct multiple comparisons, P<0.0083 and P<0.0017 are considered significance levels of 5% (*) and 1% (**), respectively.

The pFI, pGE, pMY, and pSS indicate respective types of optic disc appearances that predicted by GSAS classification formulas.

SD, standard deviation; 95% CI, 95% confidence interval; BCVA, best-corrected visual acuity; NTG, normal tension glaucoma; HTG, high tension glaucoma; IOP, intraocular pressure; MD, visual field mean deviation; PSD, visual field pattern standard deviation; pGE, formula-predicted focal ischemia; pMY, formula-predicted myopic glaucomatous; pSS, formula-predicted senile sclerotic; pGE, formula-predicted generalized enlargement.

**Fig 3:**
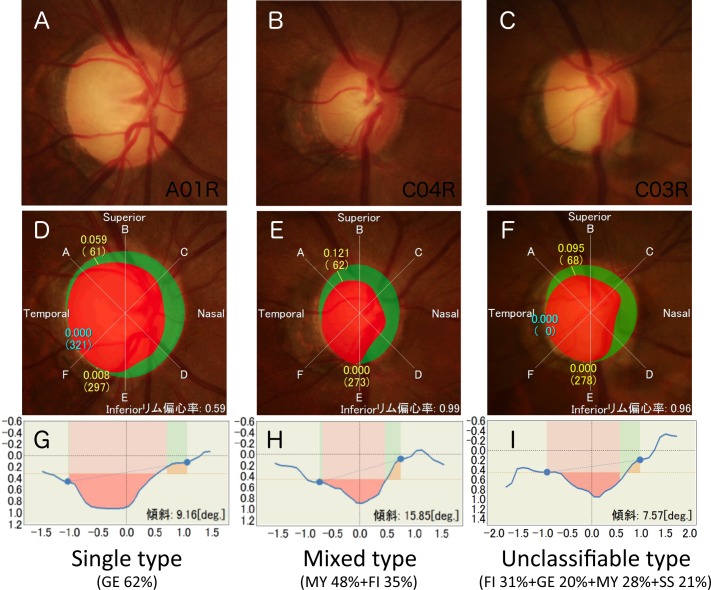
The representative optic discs have various mixing rates. The color fundus photographs (**A, B, C**), image analysis results (**D, E**, **F**), and depth maps in the horizontal meridian (**G, H, I**) from cases 1 (**A, D, G**), 2 (**B, E, H**), and 3 (**C, F, H**) are shown. Based on the discrimination formulas, cases 1, 2, and 3 are predicted to have 62% probability of GE elements, 48% and 35% probabilities of MY and FI elements, respectively, and 31%, 20%, 28%, and 21% probabilities of FI, GE, MY, and SS elements, respectively. Based on the definitions described in the Methods, cases 1, 2, and 3 are classified as having single, mixed, and unclassifiable types of optic discs, respectively.

Based on the mixing rate of the four optic disc appearances, the 187 eyes were subclassified into 118 (63%) eyes with single, 30 (16%) eyes with mixed, and 39 (21%) eyes with unclassifiable types of optic discs. Among the single type, pMY (56 eyes, 47%) was the most frequent optic disc appearance. Among the mixed type, pFI+pGE (8 eyes, 27%) and pMY+pSS (8 eyes, 27%) were the most frequent optic disc appearances (Table 4). Representative cases of optic discs with various degrees of mixing are shown in Fig 3.

**Table 4:** Distributions of single, mixed, and unclassifiable types of optic disc appearances among subjects.

Type	n (%)
Single type	118 (63.1)
Mixed type	30 (16.0)
Unclassifiable type	39 (21.0)
Breakdown of single type (n = 118)	
pFI	23 (19.5)
pGE	24 (20.3)
pMY	56 (47.5)
pSS	15 (12.7)
Breakdown of mixed type (n = 30)	
pFI+pGE	8 (26.7)
pFI+pMY	3 (10.0)
pFI+pSS	3 (10.0)
pGE+pMY	2 (6.7)
pGE+pSS	6 (20.0)
pMY+pSS	8 (26.7)

The single type is defined as an optic nerve head of one element with a probability of 60% or more. The mixed type is defined as an optic disc with more than one element and a probability of 60% or more, but either pair of the top two elements has a probability of 80% or more.

The unclassifiable type is defined as an optic disc other than the single and mixed types.

pFI, pGE, pMY, and pSS indicate respective types of optic disc appearances predicted by the Glaucoma Stereo Analysis Study classification formulas. pGE, formula-predicted focal ischemia; pMY, formula-predicted myopic glaucomatous; pSS, formula-predicted senile sclerotic; pGE, formula-predicted generalized enlargement.

## Discussion

Discrimination analysis showed six of 38 parameters selected for the GSAS classification model of different optic disc appearances. Rim-decentering and disc tilt angle, the novel parameters that we defined for the GSAS[7, 22], also were among the six parameters. By comparing the predicted optic disc appearances (Table 2), a thicker temporal rim-disc ratio and a larger rim decentering absolute value represented the FI disc; a deeper cup depth and flat disc tilt angle represented the GE disc; thinner temporal and thicker nasal rim-disc ratios, larger height variation of the contour, and larger temporal disc tilt represented the MY disc; and a flat or even nasal disc tilt represented the SS disc. These morphologic characteristics agreed well with the features of each optic disc appearance described by Nicolela and Drance as indicated in the Methods section[10]. In the current study, three graders established the true classification of the optic disc (“gold standard”) as described in the Methods section. All graders were experienced with this classification method through their previous works[15–18]; the agreement among the three graders was identical or superior to that reported previously[23]. Collectively, in addition to indicating good prediction of different optic discs by the GSAS classification formulas, the results indicated a suitable establishment of the gold standard by the graders.

In the current study, different optic disc appearances were predicted with AUC values of 0.86–0.93 by the established formulas (Fig 2); the predictability was highest with the MY disc and the lowest with the SS disc. Four of the six optic disc parameters were useful to separate the MY disc from other disc types, whereas the disc tilt angle was virtually the only determinant of the SS discs (Table 2). The flat disc angle also was a determinant of the GE disc, which explained the relatively poor separation between the SS and GE discs seen in the canonical plot (Fig 1). Other than the optic disc shape, the SS discs were characterized by surrounding PPA and choroidal sclerosis[10]; thus, use of another topographic parameter to target structures outside the optic disc using a fundus camera or tomographic parameters on optical coherence tomography[24–26] can improve discrimination of the SS disc but must be tested. Agreement among the graders was relatively poor with the SS disc compared with the other optic disc types (see Methods section), and discriminating the SS disc might be difficult.

The demographic parameters differed among the predicted disc types (Table 3); the age was younger in the pMY group and older in the pSS group; the refractive error was highly myopic in the pMY group; the rates of NTG were higher in the pFI and pMY groups and the rate of HTG was higher in the pGE group; and the baseline IOP was higher in the pGE group and lower in the pFI group. Accordingly, the current study reproduced the findings of previous reports and supported the previous conclusions that different optic disc appearances showed different clinical phenotypes of glaucoma[3, 10–18]. Previous studies have assessed the possible correlations between the optic disc appearances and various clinical factors by considering the optic disc types as categorical variables[3, 10–18]. Our methods provided the degree of mixing rate numerically (Table 4, Fig 3) and, therefore, enabled considering each optic disc as a continuous variable in future clinical study.

Although the formulas can provide the computerized classifications of the optic disc appearances, the technique requires the examiner-determined optic disc and cup margins. To apply our technique, the examiner needs a clear understanding of the criteria for the rim and cup[6]. In a few patients, PPA, for example, reduced the color contrast of Elschnig’s scleral ring, which resulted in a fuzzy image and difficulty determining the contour line of the disc edge. Nevertheless, even in such cases, a stereoscopic image provides more information than a monoscopic image and should lead to more accurate diagnoses. The formulas established originated from the particular stereo-fundus camera; thus, other coefficients were required when the other devices were used for classification. This study also was limited by being hospital-based and retrospective, although it included a relatively high number of patients. To validate further the classification methods reported in this study, we are now preparing another dataset and will report the validation study results in the near future.

Collectively, using six optic disc topography parameters obtained by stereo fundus camera, the GSAS classification formulas enabled prediction and quantification of each component of the different optic disc appearances in each eye and can be a novel way to describe GON.

## Supporting information

S1 File The GSAS classification formulas. (PDF)

S2 File Dataset underlying the findings described in this manuscript. (PDF)
