# Supplementary material for: Differentiation of glaucomatous optic discs with different appearances using optic disc topography parameters: The Glaucoma Stereo Analysis Study
Source: PLoS One. 2017 Feb 8;12(2):e0169858. doi: 10.1371/journal.pone.0169858 (PMC5298323; doi:10.1371/journal.pone.0169858)
Supplement: S1 File — (PDF) [file pone.0169858.s001.pdf]

S1 File. The GSAS classification formulas

SqDist[0]=655.4419*A*A+4.5344*B*A+342.1453*B*B+20.8175*C*A+288.9204*C*B+305.7983*C*C+11.4839*D*A-36.5454*D*B-101.8993*D*C+28.0598*D*D+0.0085*E*A-0.5685*E*B+1.5460*E*C-0.5787*E*D+0.01534*E*E+48.5341*F*A+13.8388*F*B+8.3993*F*C-1.2033*F*D+0.0050*F*E+15.1007*F*F

SqDist[FI]=SqDist[0]+(-179.6581*A)+(-174.4897*B)+(-145.3251*C)+4.8872*D+(-0.1094*E)+(-26.0264*F)+46.9709

SqDist[GE]=SqDist[0]+(-141.1148*A)+(-154.7610*B)+(-164.3586*C)+6.7265*D+(-0.1181*E)+(-20.3744*F)+41.0876

SqDist[MY]=SqDist[0]+(-100.7481*A2)+(-181.7966*B2)+(-140.6547*C)+(-1.324*D)+(-0.3071*E)+(-19.3334*F)+43.7991

SqDist[SS]=SqDist[0]+(-110.1316*A)+(-166.6384*B)+(-120.1344*C)+(-2.6073*D2)+0.0958*E+(-17.6196*F)+33.7867

Prob{FI}=1/(1+Exp(-0.5*(SqDist[GE]-SqDist[FI]))+Exp(-0.5*(SqDist[MY]-SqDist[FI]))+Exp(-0.5*(SqDist[SS]-SqDist[FI])))

Prob{GE}=1/(1+Exp(-0.5*(SqDist[FI]-SqDist[GE]))+Exp(-0.5*(SqDist[MY]-SqDist[GE]))+Exp(-0.5*(SqDist[SS]-SqDist[GE])))

Prob{MY}=1/(1+Exp(-0.5*(SqDist[FI]-SqDist[MY]))+Exp(-0.5*(SqDist[GE]-SqDist[MY]))+Exp(-0.5*(SqDist[SS]-SqDist[MY])))

Prob{SS}=1/(1+Exp(-0.5*(SqDist[FI]-SqDist[SS]))+Exp(-0.5*(SqDist[GE]-SqDist[SS]))+Exp(-0.5*(SqDist[MY]-SqDist[SS])))

Prob{FI}+Prob{GE}+Prob{MY}+Prob{SS}=1

A, RIM_DISC_RATIO_SECTION_1

B, RIM_DISC_RATIO_SECTION_4

C, MEAN_CUP_DEPTH

D, HEIGHT_VARIATION_CONTOUR

E, DISC_TILT_ANGLE

F, RIM_DECENTERING (ABS)

SqDist, square distance; Prob, probability.
