# Supplementary material for: Differentiation of glaucomatous optic discs with different appearances using optic disc topography parameters: The Glaucoma Stereo Analysis Study
Source: PLoS One. 2017 Feb 8;12(2):e0169858. doi: 10.1371/journal.pone.0169858 (PMC5298323; doi:10.1371/journal.pone.0169858)
Supplement: S2 File — (PDF) [file pone.0169858.s002.pdf]

S2 file. Dataset underlying the findings described in this manuscript.

disc type	age (y)	sex (m/f)	HT (y/n)	DM (y/n)	HL (y/n)	poag/ ntg	baseline iop (mmHg)	iop (mmHg)	meds	MD	PSD	MD slope (dB/y)	RIM_DISC_RATIO_SECTION_1	RIM_DISC_RATIO_SECTION_4	MEAN_CUP_DEPTH	HEIGHT_VARIATION_CONTOUR	DISC_TILT_ANGLE	RIM_DECENTERING_(ABS	Prob{FI}	Prob{GE}	Prob{MY}	Prob{SS}	Pred disc type
GE	63	m	n	n	n	ntg	12	11	1	−1.6	6.23	0.26	0.014	0.149	0.299	0.405	9.16	0.586	0.08	0.62	0.23	0.07	GE
GE	55	m	n	n	n	poag	28	13	0	−5.09	9.09	0.09	0.079	0.111	0.254	0.427	6.284	0.595	0.29	0.58	0.05	0.08	GE
MY	47	f	n	y	n	ntg	15.3	13	1	−0.62	5.35	0.55	0.124	0.214	0.223	0.641	15.44	0.258	0.42	0.21	0.28	0.08	FI
MY	68	f	n	n	n	ntg	16.3	16	1	−3.95	13.01	0.04	0.107	0.152	0.182	0.503	12.42	0.349	0.39	0.29	0.16	0.16	FI
MY	66	f	y	y	n	ntg	16	17	1	−3.31	10.09	0.18	0.08	0.182	0.222	0.959	20.61	0.259	0.05	0.06	0.78	0.10	MY
FI	71	f	y	n	n	ntg	13	15	2	0.02	4.05	0.13	0.044	0.215	0.197	0.351	−7.47	0.459	0.11	0.13	0.04	0.72	SS
MY	70	m	y	n	n	ntg	19	16	2	−3.71	6.6	−0.15	0.058	0.184	0.178	0.877	29.76	0.38	0.02	0.02	0.94	0.02	MY
MY	50	f	y	n	n	ntg	18	13	2	−6.02	13.24	0.51	0.1	0.238	0.215	1.053	30.75	0.692	0.12	0.01	0.86	0.01	MY
GE	71	m	y	n	n	ntg	15.8	11	1	0.17	2.86	1.09	0.082	0.178	0.204	0.48	7.18	0.051	0.11	0.29	0.19	0.41	SS
MY	48	m	n	n	n	ntg	15.5	17	1	−2.19	3.42	−0.18	0.028	0.245	0.087	0.871	31.86	0.642	0.01	0.00	0.97	0.02	MY
SS	69	f	y	y	n	ntg	13.3	18	1	−2.33	4.71	−0.02	0.149	0.155	0.171	0.883	3.46	0.159	0.24	0.08	0.05	0.62	SS
GE	73	f	n	y	n	ntg	14.5	15	0	−3.55	3.15	0.16	0.083	0.134	0.271	0.399	2.548	0.128	0.10	0.68	0.05	0.17	GE
MY	70	f	y	n	n	ntg	12.6	14	1	−0.61	2.19	−0.11	0.101	0.186	0.281	0.634	16.48	0.512	0.39	0.34	0.24	0.03	FI
GE	70	m	n	y	n	poag	21.3	19	2	−10.1	13.73	−1.86	0.114	0.109	0.204	0.249	−3.07	0.297	0.29	0.50	0.01	0.20	GE
MY	68	f	y	n	n	poag	27	15	1	−0.75	7.69	0.2	0.028	0.257	0.145	1.103	28.14	0.798	0.01	0.00	0.97	0.02	MY
MY	31	m	n	n	n	poag	19.3	18	1	−2.38	6.17	0.29	0.04	0.098	0.194	0.3	2.601	0.4	0.09	0.45	0.07	0.40	GE
MY	63	m	y	n	n	poag	21	18	3	−5.54	9.64	−0.84	0.003	0.195	0.213	1.03	23.96	0.586	0.00	0.01	0.95	0.03	MY
SS	53	m	n	n	n	ntg	14.4	16	1	−1.47	3.81	0.08	0.073	0.095	0.199	0.365	−6.69	0.121	0.04	0.27	0.01	0.68	SS
MY	63	f	n	y	n	ntg	13	13	1	−0.4	1.94	0.11	0.028	0.261	0.19	0.741	18	0.191	0.01	0.02	0.87	0.11	MY
GE	68	m	y	n	n	ntg	16	14	2	0.42	4.3	0.35	0.118	0.128	0.272	0.448	8.171	0.16	0.21	0.68	0.04	0.06	GE
MY	66	f	n	n	n	ntg	18.7	18	1	−2.28	7.48	0.28	0.001	0.213	0.149	0.692	16.84	0.041	0.00	0.01	0.74	0.24	MY
MY	67	m	n	n	n	ntg	13.3	13	0	−1.22	2.84	−0.02	0.019	0.194	0.235	0.661	17.43	0.063	0.01	0.07	0.80	0.12	MY
FI	61	f	n	n	n	ntg	15	13	1	−4.37	3.87	0.12	0.153	0.216	0.062	0.295	−9.04	0.327	0.41	0.03	0.00	0.56	SS
SS	74	m	y	y	n	ntg	12.3	12	2	−4.11	6.65	0.05	0.027	0.241	0.121	0.436	3.974	0.496	0.07	0.04	0.22	0.68	SS
FI	80	m	n	n	n	poag	34	15	2	−8.99	10.2	−0.47	0.074	0.163	0.251	0.436	0.4	0.689	0.42	0.40	0.04	0.14	FI
SS	77	f	n	n	n	ntg	13.7	11	2	−8.28	9.2	−0.16	0.032	0.116	0.204	0.381	−0.17	0.32	0.05	0.30	0.07	0.59	SS
MY	77	f	y	n	n	ntg	15.5	18	1	0.07	2.91	0.55	0.039	0.218	0.194	0.615	17.61	0.523	0.08	0.07	0.76	0.10	MY
GE	80	f	y	y	n	ntg	14	12	2	−11.2	16.12	−0.14	0.044	0.082	0.259	0.431	−13.7	0.628	0.08	0.38	0.01	0.53	SS
MY	57	f	n	y	n	poag	19.7	20	0	1.65	2.07	0.3	0.019	0.301	0.211	1.053	20.16	0.49	0.01	0.00	0.95	0.04	MY
MY	58	f	y	n	y	ntg	13	15	1	−10.9	17.51	0.2	0.099	0.238	0.138	0.707	11.76	0.745	0.57	0.04	0.23	0.17	FI
MY	52	f	n	n	n	ntg	14	14	3	−6.82	9.32	−0.14	0.037	0.154	0.341	1.213	29.81	0.637	0.01	0.03	0.95	0.00	MY
MY	54	f	n	n	n	ntg	16	12	3	−9.38	18.31	−0.18	0.024	0.272	0.189	0.942	30.09	0.501	0.00	0.00	0.98	0.01	MY
MY	50	m	n	n	n	poag	20	12	2	−3.56	4.21	−0.42	0.12	0.202	0.237	0.743	21.54	0.602	0.54	0.13	0.31	0.02	FI
GE	67	f	n	n	y	ntg	16	16	2	−6.03	10.34	−0.48	0.184	0.156	0.167	0.27	8.634	0.273	0.80	0.17	0.01	0.02	FI
MY	42	m	n	n	n	ntg	16	14	1	−0.57	2.86	−0.02	0.133	0.218	0.205	0.673	14.78	0.055	0.32	0.19	0.33	0.16	MY
SS	71	f	n	n	y	ntg	17	15	3	−4.67	6.34	−0.42	0.04	0.24	0.148	0.391	−6.94	0.353	0.04	0.03	0.04	0.89	SS
SS	68	m	n	n	n	poag	24	15	3	−4.71	9.16	0.11	0.042	0.153	0.133	0.152	2.309	0.305	0.09	0.22	0.08	0.61	SS
SS	67	m	n	n	n	poag	22	13	4	−6.55	12.23	0.05	0.035	0.197	0.126	0.564	15.84	0.132	0.02	0.04	0.60	0.34	MY
FI	64	f	y	n	n	ntg	15	11	1	−0.8	4.45	−0.23	0.119	0.156	0.359	0.473	7.187	0.594	0.38	0.60	0.02	0.01	GE
MY	60	m	n	n	n	poag	21	18	2	−6.6	8.97	0.14	0.096	0.157	0.351	0.386	9.04	0.294	0.14	0.81	0.04	0.01	GE
MY	50	m	n	n	n	ntg	18	14	1	−5.04	8.97	−0.12	0.054	0.237	0.299	0.406	8.704	0.124	0.09	0.49	0.33	0.10	GE
MY	58	m	y	n	n	ntg	14	11	3	−4.58	5.84	−0.28	0.097	0.336	0.131	0.319	2.219	0.288	0.40	0.05	0.14	0.41	SS
GE	75	m	y	n	y	ntg	17	8	0	−8.68	13.1	−0.12	0.101	0.134	0.254	0.627	−17.6	0.225	0.06	0.14	0.00	0.80	SS
FI	56	f	n	n	n	poag	21	17	0	−5.78	13.45	−0.03	0.037	0.2	0.201	0.411	0.924	0.536	0.15	0.20	0.15	0.50	SS
FI	68	f	n	n	n	ntg	14	14	1	−3.35	5.69	−0.11	0.207	0.174	0.111	0.329	−1.43	0.06	0.75	0.08	0.00	0.16	FI
MY	55	f	n	n	n	ntg	14	12	1	−8.73	14.26	−0.16	0.019	0.179	0.186	0.409	7.571	0.956	0.31	0.20	0.28	0.21	FI
MY	57	f	y	n	n	ntg	16	14	2	−2.35	11.07	0.28	0.04	0.192	0.182	0.576	15.85	0.993	0.35	0.10	0.48	0.08	MY
GE	77	m	n	n	n	ntg	15	11	2	−11.8	8.28	−0.45	0.082	0.157	0.357	0.411	7.867	0.834	0.36	0.61	0.03	0.01	GE
SS	71	f	n	n	n	ntg	17	13	1	−2.06	2.48	−0.72	0.105	0.134	0.196	0.473	8.283	0.708	0.63	0.25	0.05	0.08	FI
MY	59	f	n	n	n	ntg	18	16	3	−6.65	7.6	−0.79	0.003	0.28	0.159	1.111	42.38	0.396	0.00	0.00	1.00	0.00	MY
GE	65	m	n	n	n	poag	22	17	2	−1.78	2.47	0.23	0.123	0.111	0.34	0.413	−3.4	0.071	0.10	0.85	0.01	0.05	GE

SS	75	f	y	y	n	ntg	13	12	1	−0.33	2.93	0.05	0.08	0.226	0.167	0.353	−6.79	0.936	0.68	0.07	0.01	0.23	FI
MY	52	m	n	n	n	poag	20	14	1	−4.3	9.98	0.03	0.007	0.294	0.147	0.779	30.67	0.718	0.01	0.00	0.99	0.01	MY
GE	68	f	n	n	n	ntg	14	12	2	−11.3	15.52	−0.99	0.006	0.261	0.133	0.342	2.346	0.618	0.07	0.04	0.25	0.63	SS
MY	68	m	n	n	n	ntg	16	17	2	−11	12.16	−0.4	0.014	0.251	0.175	0.88	30.22	0.425	0.00	0.00	0.98	0.01	MY
GE	64	m	n	n	n	poag	25	24	3	−5.48	5.58	0.02	0.075	0.126	0.531	1.275	−22.9	0.662	0.08	0.67	0.01	0.24	GE
GE	74	m	n	n	n	ntg	18	13	3	−9.92	6.93	−0.35	0.111	0.071	0.243	1.002	20.52	0.775	0.45	0.24	0.26	0.05	FI
FI	60	f	n	n	n	poag	25	12	2	−8.82	11.94	0.02	0.075	0.137	0.232	1.087	16.32	0.337	0.05	0.09	0.62	0.24	MY
MY	49	m	n	n	n	ntg	18	18	2	−8.19	14.27	−0.39	0.082	0.193	0.185	0.607	6.419	0.345	0.20	0.15	0.20	0.45	SS
GE	68	m	n	n	n	poag	22	16	2	−2.64	4.24	−0.1	0.09	0.064	0.289	0.194	3.78	0.457	0.13	0.85	0.01	0.02	GE
GE	73	m	n	n	n	poag	24	13	2	−11.7	9.55	1.61	0.07	0.129	0.197	0.374	7.038	0.974	0.62	0.26	0.05	0.07	FI
SS	46	m	n	n	n	ntg	12	12	1	−11.1	10.12	0.19	0.04	0.069	0.697	1.997	−31.4	0.04	0.00	0.34	0.00	0.65	SS
FI	67	f	y	n	n	ntg	18	16	3	−6.96	13.17	−0.91	0.045	0.125	0.266	0.453	−0.96	0.683	0.19	0.54	0.05	0.22	GE
MY	66	f	n	n	n	ntg	14	12	2	−4.82	7.69	−0.45	0.09	0.194	0.243	0.627	17.39	0.567	0.37	0.23	0.35	0.04	FI
FI	58	f	n	n	y	ntg	14	16	1	−5.97	13.2	−0.35	0.101	0.283	0.182	0.335	3.09	0.399	0.57	0.13	0.10	0.20	FI
MY	49	m	n	n	n	ntg	16	14	1	−4.09	10.98	−0.09	0.003	0.207	0.15	0.866	13.73	0.892	0.04	0.01	0.70	0.24	MY
FI	63	m	n	n	n	ntg	8	8	1	−1.11	9.34	−0.44	0.132	0.27	0.179	0.379	2.827	0.554	0.83	0.07	0.03	0.07	FI
FI	68	f	n	y	n	ntg	18	16	1	−3.48	3.93	−0.34	0.069	0.107	0.135	0.309	6.422	0.874	0.54	0.22	0.05	0.18	FI
GE	71	f	n	n	n	poag	20	17	1	−4.07	3.49	−0.18	0.042	0.075	0.276	0.593	−11.9	0.697	0.08	0.36	0.01	0.55	SS
MY	75	m	n	n	n	ntg	15	10	1	−1.25	3.53	0.07	0.02	0.213	0.192	0.572	13.22	0.888	0.18	0.09	0.61	0.13	MY
GE	59	m	n	n	n	poag	20	15	1	−4.35	9.36	−0.48	0.067	0.118	0.285	0.271	4.007	0.439	0.14	0.78	0.03	0.05	GE
MY	61	m	n	n	n	ntg	18	16	2	−6.14	12.86	−0.37	0.097	0.243	0.133	0.511	13.48	0.342	0.32	0.08	0.36	0.24	MY
MY	61	m	n	y	n	ntg	16	14	1	−9.67	12.66	−0.01	0.1	0.254	0.154	0.884	44.14	0.203	0.01	0.00	0.99	0.00	MY
SS	71	m	n	n	y	poag	36	17	3	−8.64	11.63	−0.43	0.032	0.036	0.282	0.548	−13.3	0.029	0.00	0.25	0.00	0.74	SS
GE	68	f	n	n	n	ntg	12	10	1	−2.54	3.4	−0.1	0.08	0.141	0.248	0.326	−4.89	0.379	0.20	0.53	0.02	0.26	GE
GE	75	m	n	n	y	ntg	18	13	3	−5.44	8.84	−0.35	0.125	0.07	0.428	0.709	−17.7	0.043	0.04	0.86	0.00	0.10	GE
MY	40	m	n	y	n	ntg	16.7	14	2	−2.65	2.36	−0.03	0.029	0.262	0.186	0.77	20.96	0.066	0.00	0.01	0.91	0.07	MY
GE	76	m	n	n	n	ntg	12.5	11	1	−8.97	12.05	0	0.056	0.088	0.306	0.232	−4.56	0.627	0.12	0.82	0.01	0.05	GE
MY	51	f	n	n	n	ntg	18.5	15	4	−6.72	12.22	−0.33	0.002	0.228	0.22	0.784	18.49	0.963	0.05	0.03	0.88	0.04	MY
MY	51	f	n	n	n	ntg	14	10	1	−10.3	14.33	0.19	0.048	0.162	0.127	0.405	10.45	0.6	0.21	0.14	0.27	0.38	SS
MY	69	m	n	y	n	ntg	11	10	0	−9.4	11.01	−0.41	0.021	0.18	0.19	0.708	18.61	0.444	0.02	0.05	0.80	0.12	MY
FI	66	m	n	y	y	ntg	11.3	11	2	−3.37	6.8	−0.33	0.115	0.168	0.159	0.442	9.044	0.568	0.66	0.16	0.06	0.12	FI
MY	54	m	n	y	n	ntg	14.7	9	1	−2.29	2.3	−0.07	0.072	0.4	0.105	0.678	21.79	0.048	0.02	0.00	0.92	0.06	MY
FI	65	m	n	n	n	ntg	13.3	12	3	−8.52	14.21	−0.92	0.133	0.113	0.154	0.434	10.92	0.283	0.47	0.31	0.06	0.16	FI
MY	57	m	n	y	n	ntg	15.3	12	1	−6.31	12.59	−0.94	0.085	0.274	0.119	0.798	34.89	0.46	0.03	0.00	0.95	0.01	MY
FI	54	m	n	n	n	ntg	17	12	1	−3.43	8.57	0.24	0.171	0.212	0.146	0.386	6.879	0.161	0.75	0.12	0.03	0.11	FI
MY	37	m	n	n	n	ntg	16.5	10	2	−3.87	11.09	−0.56	0.057	0.18	0.171	0.517	13.57	0.688	0.30	0.15	0.38	0.17	MY
MY	63	f	n	n	n	ntg	16	17	0	−2.79	8.57	−0.57	0.149	0.21	0.206	0.59	15.77	0.15	0.54	0.21	0.18	0.07	FI
MY	60	f	n	n	n	ntg	14	13	1	−7.81	10.93	−0.21	0.015	0.205	0.082	0.595	12.56	0.513	0.02	0.01	0.46	0.50	SS
FI	73	f	y	y	y	ntg	15	14	2	−4.31	6.25	−0.13	0.124	0.153	0.196	0.468	15.79	0.859	0.82	0.13	0.04	0.01	FI
MY	57	f	n	y	n	ntg	16	12	0	−1.66	5.83	0.07	0.128	0.137	0.237	0.702	9.777	0.847	0.78	0.16	0.03	0.03	FI
FI	58	f	n	y	n	ntg	9	10	2	−6.56	14.85	−0.69	0.125	0.119	0.198	0.381	1.036	0.507	0.55	0.32	0.01	0.12	FI
MY	48	m	n	n	n	ntg	17.3	14	0	−5.91	11.99	−0.03	0.012	0.188	0.153	0.57	12.56	0.692	0.07	0.06	0.58	0.29	MY
FI	70	f	n	n	n	ntg	16	10	2	−8.4	13.26	−0.23	0.147	0.2	0.246	0.436	4.735	0.949	0.92	0.07	0.00	0.00	FI
MY	56	f	n	n	n	ntg	14	11	3	−9.26	13.58	−1.1	0.043	0.238	0.12	0.379	4.211	0.128	0.03	0.04	0.17	0.75	SS
SS	74	m	y	y	y	poag	35	12	2	−4.3	2.85	0.17	0.07	0.418	0.033	0.508	14.54	0.257	0.06	0.00	0.68	0.25	MY
SS	61	m	y	n	y	ntg	12	10	1	−2.71	5.94	0.09	0.111	0.109	0.171	0.351	−0.7	0.53	0.46	0.29	0.01	0.23	FI
FI	64	f	y	n	y	ntg	12.3	10	0	−1.63	3.41	−0.19	0.127	0.159	0.207	0.445	2.26	0.474	0.60	0.26	0.02	0.12	FI
MY	52	m	n	n	n	ntg	15	9	2	−4.71	11.71	−0.04	0.026	0.136	0.227	0.333	8.045	0.131	0.03	0.45	0.23	0.29	GE
MY	71	f	n	n	n	ntg	17	12	1	−8.26	11.95	−0.15	0.087	0.377	0.206	0.665	20.97	0.087	0.05	0.01	0.91	0.03	MY
MY	63	m	n	y	n	ntg	17.7	13	2	−1.8	8.28	0.07	0.031	0.208	0.216	0.798	28.38	0.632	0.02	0.02	0.95	0.01	MY
MY	61	m	n	y	y	ntg	15	8	1	−9.1	13.93	−0.25	0.011	0.192	0.155	0.592	22.23	0.387	0.01	0.03	0.88	0.08	MY
MY	69	f	n	y	n	ntg	15	11	1	−10.3	14.43	0.08	0.039	0.207	0.109	0.96	23.06	0.651	0.02	0.01	0.86	0.11	MY
MY	67	m	n	n	n	ntg	17	12	1	0.04	3.02	0.12	0.003	0.309	0.138	0.772	22.18	0.449	0.00	0.00	0.95	0.04	MY
MY	54	m	y	n	n	ntg	18	13	1	−10.9	12.19	−0.18	0.006	0.301	0.215	0.989	37.94	0.908	0.00	0.00	1.00	0.00	MY
MY	50	m	n	n	n	ntg	19	13	1	−8.89	15.22	−0.13	0.002	0.205	0.115	0.461	14.39	0.865	0.09	0.04	0.64	0.22	MY
MY	61	m	y	y	y	ntg	18	11	2	−4.11	6.6	0.03	0.033	0.233	0.122	0.755	27.03	0.144	0.00	0.01	0.94	0.05	MY
MY	51	f	y	y	n	ntg	15.7	14	1	−1.89	6.14	−0.18	0.075	0.245	0.135	0.531	15.01	0.236	0.12	0.06	0.56	0.26	MY
MY	45	m	n	n	n	ntg	12.5	7	0	−4.06	4.05	−0.49	0.145	0.282	0.17	0.468	15.47	0.039	0.52	0.12	0.27	0.10	FI

FI	56	f	n	n	n	ntg	15.3	15	0	-0.93	6.02	-0.12	0.11	0.214	0.211	0.586	18.65	0.201	0.27	0.20	0.46	0.07	MY
MY	62	m	n	y	n	ntg	13.7	12	0	-3.23	2.62	0.27	0.079	0.14	0.299	0.492	16.65	0.017	0.06	0.67	0.23	0.04	GE
MY	60	m	n	n	y	poag	30	14	3	-10.7	14	0.18	0.086	0.187	0.259	0.455	6.197	0.304	0.25	0.49	0.12	0.14	GE
FI	53	m	n	n	n	ntg	19	14	1	-2.74	7.7	0.02	0.137	0.15	0.232	0.361	4.976	0.389	0.56	0.38	0.02	0.05	FI
MY	54	m	n	n	n	ntg	11.3	9	1	-3.47	6.59	0.17	0.011	0.329	0.147	0.929	35.36	0.159	0.00	0.00	1.00	0.00	MY
MY	63	f	n	n	n	ntg	19	13	3	-9.37	12.2	-0.75	0.027	0.258	0.144	0.534	15.95	0.77	0.12	0.03	0.73	0.12	MY
MY	51	f	n	y	n	ntg	17	12	1	-4.91	8.91	-0.7	0.028	0.202	0.179	0.737	20.05	0.002	0.01	0.02	0.83	0.14	MY
SS	68	f	n	n	y	ntg	14.3	13	1	-2.54	6.46	-0.56	0.112	0.239	0.15	0.305	2.268	0.571	0.73	0.10	0.03	0.14	FI
MY	57	f	n	n	y	ntg	18.7	16	2	-2.8	2.8	-0.53	0.081	0.391	0.156	0.868	24.53	0.096	0.01	0.00	0.96	0.03	MY
MY	51	f	y	y	y	ntg	16.3	12	3	-11	14.08	-0.57	0.079	0.218	0.12	0.646	26.03	0.027	0.03	0.02	0.87	0.08	MY
MY	62	f	n	y	n	ntg	19	12	0	-5.82	10.01	-0.13	0.072	0.237	0.061	0.863	25.53	0.071	0.01	0.00	0.84	0.15	MY
MY	67	f	y	y	n	poag	20	14	1	-7.05	13.35	-0.13	0.062	0.281	0.116	0.555	7.478	0.72	0.31	0.03	0.30	0.37	SS
GE	59	f	n	n	n	ntg	12.7	13	0	-2.25	6.54	-0.15	0.128	0.102	0.225	0.25	5.778	0.02	0.19	0.72	0.02	0.08	GE
SS	74	m	n	n	n	ntg	15	11	1	-6.41	12.42	-0.09	0.033	0.209	0.164	0.36	0.165	0.827	0.30	0.13	0.11	0.46	SS
MY	70	m	n	n	n	ntg	18	13	2	-9.38	10.04	-0.12	0.032	0.297	0.036	0.742	13.32	0.43	0.02	0.00	0.50	0.48	MY
FI	63	m	n	n	n	ntg	17.3	13	1	-0.39	4.14	0.17	0.144	0.277	0.181	0.646	14.09	0.661	0.86	0.03	0.08	0.02	FI
MY	59	f	n	n	n	ntg	16	14	1	-8.49	13.86	-0.14	0.01	0.154	0.293	0.584	-12.1	0.496	0.03	0.21	0.03	0.74	SS
MY	53	m	y	n	n	ntg	15	12	1	-2.61	3.2	-0.32	0.045	0.259	0.173	0.912	24.24	0.25	0.01	0.01	0.94	0.05	MY
FI	52	m	n	n	n	ntg	13.3	14	0	-3.36	3.74	-0.49	0.123	0.169	0.235	0.387	-5.46	0.892	0.82	0.14	0.00	0.04	FI
MY	54	m	y	n	n	poag	20	13	1	-8.72	13.69	-0.05	0.017	0.228	0.111	0.615	12.75	0.105	0.01	0.01	0.50	0.48	MY
MY	63	m	y	y	y	ntg	18	13	2	-10.3	13.64	-0.07	0.01	0.138	0.438	1.408	36.1	0.054	0.00	0.02	0.98	0.00	MY
GE	50	m	n	n	n	poag	20	14	2	-5.31	7.76	-0.01	0.11	0.098	0.302	0.552	-6.23	0.512	0.28	0.59	0.01	0.12	GE
MY	67	m	n	n	n	ntg	13	12	0	-2.15	4.93	0.09	0.155	0.391	0.133	0.675	13.99	0.492	0.79	0.01	0.16	0.04	FI
MY	56	f	n	n	n	ntg	12.3	12	0	-4.17	10.32	-0.31	0.081	0.319	0.119	1.066	38.99	0.023	0.00	0.00	0.99	0.00	MY
FI	71	m	n	n	n	poag	23	15	2	-3.22	10.28	-0.07	0.032	0.215	0.077	0.339	5.828	0.643	0.13	0.04	0.19	0.65	SS
GE	64	m	y	y	n	ntg	18	11	1	-2.61	3.37	-0.04	0.063	0.171	0.242	0.724	12.64	0.722	0.29	0.22	0.37	0.12	MY
MY	48	m	n	n	n	ntg	13.7	13	0	-1.07	3.33	0.11	0.093	0.158	0.227	0.867	20.41	0.663	0.31	0.14	0.49	0.05	MY
SS	63	m	n	y	n	poag	19.3	17	0	-3.91	8.73	-0.56	0.055	0.3	0.094	0.366	9.962	0.135	0.06	0.03	0.43	0.49	SS
MY	55	f	n	n	n	ntg	16	14	0	-3.16	8.45	0.24	0.071	0.186	0.291	0.551	12.45	0.046	0.07	0.49	0.34	0.10	GE
GE	70	f	y	y	n	ntg	18	14	1	-3.48	4.71	-0.01	0.085	0.132	0.197	0.566	2.762	0.348	0.18	0.26	0.07	0.49	SS
MY	57	m	y	n	y	ntg	16	15	1	-4.58	12.09	-0.87	0.055	0.365	0.114	0.536	11.33	0.583	0.17	0.01	0.62	0.20	MY
GE	75	m	n	n	n	ntg	13.1	9	0	-2.06	5.88	-0.01	0.086	0.174	0.254	0.318	6.034	0.131	0.16	0.63	0.09	0.13	GE
FI	64	f	n	y	n	ntg	15	14	1	-4.05	8.45	-0.3	0.129	0.135	0.216	0.553	8.33	0.855	0.81	0.15	0.02	0.02	FI
MY	50	m	n	n	n	ntg	18.3	16	1	-4.9	8.08	0.85	0.006	0.151	0.223	0.739	17.92	0.487	0.02	0.08	0.79	0.11	MY
MY	48	m	n	y	n	ntg	14.6	15	2	-5.78	7.74	-0.24	0.038	0.189	0.263	0.806	18.03	0.265	0.02	0.10	0.80	0.08	MY
GE	64	m	n	y	n	ntg	13	15	1	-5.54	3.72	0.08	0.082	0.161	0.36	0.543	9.044	0.61	0.25	0.68	0.06	0.01	GE
FI	56	f	n	n	n	ntg	14	14	2	-2.41	9.19	-0.26	0.144	0.132	0.204	0.285	3.56	0.541	0.70	0.26	0.01	0.03	FI
FI	58	f	n	n	n	ntg	12	13	0	-2.6	3.96	-0.06	0.159	0.19	0.125	0.321	0.112	0.056	0.49	0.14	0.02	0.36	FI
MY	64	f	n	y	n	ntg	14	13	0	-1.37	7.71	0.48	0.081	0.253	0.209	0.542	17.11	0.189	0.13	0.12	0.65	0.09	MY
MY	67	m	n	y	y	ntg	12	14	0	-3.31	6.62	-0.06	0.016	0.314	0.151	0.682	26.65	0.512	0.01	0.00	0.97	0.01	MY
MY	62	f	n	n	n	ntg	17	14	1	-0.48	2.54	0.12	0.05	0.145	0.33	0.511	5.419	0.776	0.23	0.66	0.08	0.04	GE
GE	55	m	n	n	n	ntg	17	12	1	-5.7	11.86	0.07	0.035	0.186	0.314	0.345	8.886	0.389	0.09	0.67	0.19	0.05	GE
GE	36	f	n	n	n	ntg	14.5	13	0	-2.47	4.76	-0.19	0.061	0.08	0.334	0.205	-3.6	0.198	0.03	0.92	0.01	0.04	GE
MY	63	f	n	n	n	ntg	19	14	1	-7.41	12.14	-0.54	0.016	0.215	0.244	0.575	17.86	0.397	0.03	0.10	0.82	0.06	MY
MY	59	f	n	n	n	ntg	14.7	13	1	-4.95	9.24	-0.47	0.08	0.198	0.194	0.463	6.511	0.215	0.18	0.24	0.19	0.38	SS
FI	76	f	y	y	y	ntg	19	16	0	-6.36	10.48	0.07	0.022	0.295	0.317	0.257	-1.89	0.516	0.19	0.49	0.18	0.14	GE
SS	62	m	n	n	n	ntg	18	17	1	-7.46	12.03	0.01	0.017	0.13	0.144	0.221	4.726	0.185	0.03	0.19	0.13	0.66	SS
SS	64	m	y	n	y	ntg	15.3	16	0	-0.52	2.54	0.22	0.118	0.19	0.226	0.577	12.11	0.333	0.46	0.28	0.16	0.09	FI
MY	57	f	n	n	n	ntg	12.7	9	1	-4.42	10.4	-0.45	0.07	0.405	0.092	0.84	27.56	0.639	0.04	0.00	0.95	0.01	MY
MY	56	m	y	n	n	ntg	16.3	14	0	-1.88	2.34	0.18	0.019	0.252	0.284	1.381	34.12	0.717	0.00	0.00	1.00	0.00	MY
MY	43	f	n	n	n	ntg	13.3	13	0	-4.69	5.54	-0.37	0.046	0.273	0.091	0.68	27.32	0.216	0.01	0.00	0.94	0.05	MY
MY	64	m	y	n	y	ntg	16	10	1	-4.37	8.54	-0.04	0.036	0.338	0.122	0.676	21.09	0.348	0.01	0.00	0.92	0.06	MY
FI	68	f	n	n	n	ntg	15	12	1	-3.55	9.12	-0.03	0.081	0.192	0.19	0.32	10.36	0.87	0.71	0.17	0.08	0.04	FI
FI	56	f	n	n	n	ntg	13.6	12	0	-5.44	12.11	-0.1	0.111	0.272	0.205	0.605	14.72	0.249	0.35	0.11	0.43	0.10	MY
MY	50	m	n	n	n	poag	30	14	1	-8.51	10.05	0.12	0.014	0.357	0.095	0.737	19.09	0.03	0.00	0.00	0.88	0.12	MY
MY	39	f	n	n	n	poag	21	15	2	-7.22	11.17	-0.12	0.017	0.275	0.089	0.555	18.6	0.684	0.04	0.01	0.82	0.12	MY
FI	59	f	n	n	n	ntg	19	15	2	-3.93	7.68	-0.5	0.116	0.165	0.176	0.379	7.292	0.758	0.79	0.14	0.03	0.05	FI
MY	59	m	n	n	n	poag	25	16	3	-6.04	12.26	0.04	0.006	0.233	0.114	0.342	6.543	0.747	0.11	0.05	0.35	0.49	SS

MY	64	m	y	y	n	poag	22	15	2	−3.45	7.38	−0.65	0.004	0.089	0.291	0.479	−14	0.712	0.04	0.37	0.01	0.58 SS
MY	54	m	n	n	n	poag	24	14	3	−11.6	10.34	−0.13	0.022	0.097	0.323	0.408	7.095	0.545	0.05	0.82	0.08	0.05 GE
FI	69	m	y	n	n	ntg	15	10	1	−9.94	16.36	0.09	0.165	0.213	0.179	0.472	8.334	0.879	0.96	0.03	0.01	0.01 FI
MY	59	m	n	n	n	ntg	14	11	2	−2.1	5.41	0.11	0.124	0.355	0.068	0.545	5.926	0.582	0.65	0.01	0.10	0.24 FI
FI	69	m	n	n	n	poag	22	21	2	−9.04	14.19	−0.92	0.091	0.12	0.225	0.623	7.353	0.798	0.54	0.28	0.07	0.10 FI
MY	70	f	y	n	n	ntg	18	15	1	−1.69	5.52	0.24	0.004	0.175	0.101	0.651	14.32	0.27	0.01	0.01	0.50	0.48 MY
GE	43	m	n	n	n	poag	25	15	1	1.87	1.08	0.14	0.064	0.129	0.281	0.434	8.008	0.189	0.08	0.70	0.11	0.11 GE
MY	53	m	n	n	n	ntg	13	10	2	0.51	1.62	−0.25	0.12	0.195	0.139	0.384	5.675	0.319	0.50	0.15	0.06	0.28 FI
FI	74	f	y	n	n	ntg	14	17	0	−2.03	1.84	−0.2	0.114	0.188	0.255	0.617	14.44	0.928	0.80	0.13	0.06	0.01 FI
GE	69	f	n	n	n	ntg	19	14	1	−3.11	5.09	−0.23	0.046	0.097	0.253	0.332	4.984	0.761	0.22	0.65	0.05	0.08 GE
GE	63	f	n	n	n	ntg	16	15	1	−1.01	2.04	−0.1	0.079	0.111	0.253	0.73	6.071	0.762	0.38	0.36	0.09	0.16 FI
GE	62	m	y	n	n	poag	20	14	3	−2.01	2.5	−0.08	0.098	0.171	0.335	0.941	−9.09	0.391	0.17	0.29	0.03	0.51 SS
SS	80	m	y	y	y	ntg	18	11	2	−2.23	2.6	0.19	0.016	0.256	0.077	0.281	−4.4	0.534	0.03	0.01	0.05	0.91 SS
FI	66	f	y	n	n	ntg	13	7	1	−7.63	13.19	0.89	0.015	0.268	0.201	0.247	−2.66	0.599	0.16	0.16	0.16	0.52 SS
GE	63	f	n	n	n	ntg	17	18	3	−2.57	6.34	−0.1	0.069	0.093	0.316	0.434	0.007	0.669	0.18	0.75	0.02	0.05 GE
GE	67	f	y	y	n	ntg	18	17	1	0.83	1.64	−0.55	0.136	0.174	0.237	0.433	7.764	0.307	0.54	0.37	0.04	0.06 FI
GE	70	m	y	y	n	ntg	18	13	1	−1.17	1.98	0.2	0.1	0.153	0.286	0.341	3.414	0.053	0.13	0.75	0.04	0.09 GE
GE	70	f	n	n	y	ntg	14	16	0	−3.09	2.51	−0.05	0.072	0.07	0.291	0.333	−2.48	0.258	0.06	0.82	0.01	0.11 GE
MY	45	m	n	n	n	ntg	14	17	0	−0.68	1.71	0.21	0.027	0.174	0.23	0.476	7.155	0.605	0.14	0.30	0.31	0.25 MY
GE	63	f	n	n	n	ntg	17	14	3	−1.07	2.22	−0.09	0.095	0.111	0.162	0.297	−3.95	0.313	0.19	0.28	0.01	0.52 SS
